# Novel ethyl methanesulfonate (EMS)-induced null alleles of the *Drosophila* homolog of *LRRK2* reveal a crucial role in endolysosomal functions and autophagy *in vivo*

**DOI:** 10.1242/dmm.017020

**Published:** 2014-10-02

**Authors:** Mark W. Dodson, Lok K. Leung, Mohiddin Lone, Michael A. Lizzio, Ming Guo

**Affiliations:** 1 Department of Neurology, University of California, Los Angeles, CA 90095, USA.; 2 Molecular Biology Institute, University of California, Los Angeles, CA 90095, USA.; 3 Brain Research Institute, The David Geffen School of Medicine, University of California, Los Angeles, CA 90095, USA.; 4 Molecular and Medical Pharmacology, University of California, Los Angeles, CA 90095, USA.

**Keywords:** LRRK2, Lysosome, Parkinson’s disease, *Drosophila*, Autophagy, Endosomes, Rab7, Rab9

## Abstract

Mutations in *LRRK2* cause a dominantly inherited form of Parkinson’s disease (PD) and are the most common known genetic determinant of PD. Inhibitor-based therapies targeting LRRK2 have emerged as a key therapeutic strategy in PD; thus, understanding the consequences of inhibiting the normal cellular functions of this protein is vital. Despite much interest, the physiological functions of *LRRK2* remain unclear. Several recent studies have linked the toxicity caused by overexpression of pathogenic mutant forms of *LRRK2* to defects in the endolysosomal and autophagy pathways, raising the question of whether endogenous *LRRK2* might play a role in these processes. Here, we report the characterization of multiple novel ethyl methanesulfonate (EMS)-induced nonsense alleles in the *Drosophila LRRK2* homolog, *lrrk*. Using these alleles, we show that *lrrk* loss-of-function causes striking defects in the endolysosomal and autophagy pathways, including the accumulation of markedly enlarged lysosomes that are laden with undigested contents, consistent with a defect in lysosomal degradation. *lrrk* loss-of-function also results in the accumulation of autophagosomes, as well as the presence of enlarged early endosomes laden with mono-ubiquitylated cargo proteins, suggesting an additional defect in lysosomal substrate delivery. Interestingly, the lysosomal abnormalities in these *lrrk* mutants can be suppressed by a constitutively active form of the small GTPase *rab9*, which promotes retromer-dependent recycling from late endosomes to the Golgi. Collectively, our data provides compelling evidence of a vital role for *lrrk* in lysosomal function and endolysosomal membrane transport *in vivo*, and suggests a link between *lrrk* and retromer-mediated endosomal recycling.

## INTRODUCTION

Parkinson’s disease (PD) is a common and devastating neurodegenerative movement disorder. The leucine-rich repeat kinase 2 (*LRRK2*) gene is an important therapeutic target for PD because it is the most common known genetic determinant of the disease (Dawson et al., 2010; Kett and Dauer, 2012). *LRRK2* mutations were originally identified as the causative factor in dominantly inherited forms of PD linked to the *PARK8* locus (Paisán-Ruíz et al., 2004; Zimprich et al., 2004) and, more recently, sequence variation at the *LRRK2* locus has been associated with an increased risk of developing sporadic PD in genome-wide association studies (Satake et al., 2009; Simón-Sánchez et al., 2009). *LRRK2* encodes a large multi-domain protein characterized by leucine-rich repeats, a GTPase domain and a kinase domain (Bosgraaf and Van Haastert, 2003). The cellular functions of *LRRK2* remain unclear because it has been linked to multiple diverse cellular processes, including mitochondrial function (Smith et al., 2005), regulation of transcription (Kanao et al., 2010) and translation (Gehrke et al., 2010; Imai et al., 2008; Martin et al., 2014), Golgi protein sorting (Sakaguchi-Nakashima et al., 2007), apoptosis (Ho et al., 2009), and regulation of the dynamics of actin (Jaleel et al., 2007; Parisiadou et al., 2009) and microtubules (Gandhi et al., 2008; Gillardon, 2009; Kett et al., 2012; Lin et al., 2009).

Understanding the normal cellular functions of *LRRK2* is vital because the mechanisms mediating the pathogenicity of mutant forms of *LRRK2* are likely to be related to the physiological functions of the wild-type protein. Moreover, although inhibitor-based therapies targeting LRRK2 have emerged as a prime therapeutic target in PD (Lee et al., 2012), the effects of inhibiting endogenous LRRK2 are not clear. Several recent studies have suggested a role for *LRRK2* and its homologs in lysosomal function and in membrane trafficking in the endolysosomal and autophagy pathways; however, these interpretations derive largely from studies of overexpression of *LRRK2* and its pathological mutant forms. This raises the question of whether endogenous *LRRK2* plays a role in endolysosomal processes under physiological conditions.

Despite the diversity of cellular functions to which *LRRK2* has been linked, *LRRK2* and its homologs have been consistently found to localize to intracellular membranes in the endolysosomal pathway (Alegre-Abarrategui et al., 2009; Berger et al., 2010; Biskup et al., 2006; Hatano et al., 2007; Higashi et al., 2009; Shin et al., 2008). We have previously reported that the protein encoded by the *Drosophila* homolog of *LRRK2*, *lrrk*, localizes specifically to the membranes of late endosomes and lysosomes, and to a lesser extent to early endosomes, *in vivo* (Dodson et al., 2012). We have found that Lrrk physically binds to the late endosomal protein Rab7, and overexpression of a PD-causing mutant form of *lrrk* results in Rab7-mediated lysosomal positioning defects (Dodson et al., 2012). Interestingly, mammalian LRRK2 has recently been found to interact physically with Rab7L1 (Beilina et al., 2014; MacLeod et al., 2013), a homolog of Rab7 (Shimizu et al., 1997). Via this interaction, LRRK2 has been linked in overexpression-based studies to retromer-dependent endosome-to-Golgi membrane transport via interactions with Vps35 (MacLeod et al., 2013), and to Rab7L1-dependent lysosomal clearance of Golgi-derived vesicles (Beilina et al., 2014). Moreover, multiple studies have reported that overexpression of either wild-type or pathogenic mutant forms of *LRRK2* result in the accumulation of aberrant lysosomal and autophagosomal structures (Alegre-Abarrategui et al., 2009; Bravo-San Pedro et al., 2012; Gómez-Suaga et al., 2012; MacLeod et al., 2006; MacLeod et al., 2013; Orenstein et al., 2013; Plowey et al., 2008; Ramonet et al., 2011).

**TRANSLATIONAL IMPACT**

**Clinical issue**

Parkinson’s disease (PD) is a devastating neurodegenerative movement disorder with no cure. Mutations in the leucine-rich repeat kinase 2 (*LRRK2*) gene cause a dominantly inherited form of PD. In addition, sequence variation at the *LRRK2* locus has been associated with risk for sporadic PD, making *LRRK2* the most common genetic determinant of PD. Despite intense research efforts, the normal cellular functions of *LRRK2* remain unclear. Understanding the *in vivo* functions of *LRRK2* is crucial because *LRRK2* inhibition has emerged as a prime therapeutic strategy for PD.

**Results**

In this study, multiple nonsense mutations in the *Drosophila LRRK2* homolog *lrrk* were characterized, demonstrating that these novel *lrrk* mutants show multiple defects in the endolysosomal and autophagy pathways. These include accumulation of markedly enlarged lysosomes containing undigested cellular contents, enlarged early endosomes laden with monoubiquitylated cargo proteins, and autophagosomes. Interestingly, the lysosomal defects in these *lrrk* mutant flies can be suppressed by enhancing the expression of *rab9*, a gene encoding a small GTPase with which Lrrk colocalizes and physically binds to at the late endosomal membrane. This suggests that augmenting Rab9 activity, which is involved in retrograde transport from late endosomes to the trans-Golgi network, might bypass a need for *lrrk*, supporting a role for *lrrk* in the Rab9-trafficking pathway.

**Implications and future directions**

These results demonstrate the crucial role played by the *Drosophila LRRK2* homolog in lysosomal transport and in cargo trafficking to the lysosome in the endocytic pathway. The interactions between Lrrk and Rab9 suggest that a defect in *rab9*-dependent endosome-to-Golgi transport might underlie lysosome dysfunction in *lrrk* mutant flies. The findings suggest that efforts to target *LRRK2* activity therapeutically will need to be specifically aimed at mutant *LRRK2* alleles. Reducing the activity of endogenous wild-type *LRRK2* might in fact result in cellular toxicity due to impaired lysosomal function and endolysosomal membrane transport.

Although these studies suggest a role for *LRRK2* in endolysosomal processes, what is lacking is strong evidence for a physiological role for *LRRK2* in endolysosomal functions *in vivo*. The strongest evidence to date comes from analysis of renal epithelial cells in *LRRK2* knockout mice, showing changes in the numbers of autophagosomes and autolysosomes, as well as accumulation of lipofuscin granules, which can be suggestive of lysosome dysfunction (Tong et al., 2012). Loss-of-function of *Drosophila lrrk* has been reported to cause defects in synaptic vesicle endocytosis at the larval neuromuscular junction (NMJ) that is associated with decreased uptake of the tracer FM1-43, which is internalized via endocytosis (Matta et al., 2012). However, this experimental system is limited because the NMJ lacks the resolution for detailed subcellular analysis. In our previous work, we used the best-characterized *Drosophila lrrk* loss-of-function allele, which results from insertion of a PiggyBac transposable element within a *lrrk* intron (Imai et al., 2008; Lee et al., 2007; Matta et al., 2012; Wang et al., 2008), and found only mild defects in the endolysosomal pathway under endogenous conditions. Our data suggested that endogenous *lrrk* does play a role in regulating lysosome positioning, but only in a sensitized genetic system in which a constitutively active form of *rab7* is expressed (Dodson et al., 2012).

Because proper functioning of the endolysosomal pathway is crucial for cellular homeostasis, it is puzzling that stronger phenotypes have not been detected in the context of loss-of-function of *LRRK2* or its homologs, if indeed it plays a vital role in these processes. Thus, we hypothesized that the published *Drosophila lrrk* allele might not uncover the full range of phenotypes caused by *lrrk* loss-of-function. To address this issue, we have generated multiple novel alleles of *Drosophila lrrk* with in-frame stop codons, which should thus function as true null alleles. We report here striking phenotypes in both the endolysosomal and autophagy pathways in these flies.

## RESULTS

### Novel *lrrk* nonsense alleles cause reduced female fertility associated with premature caspase-dependent apoptosis of follicle cells

**Fig. 1.**
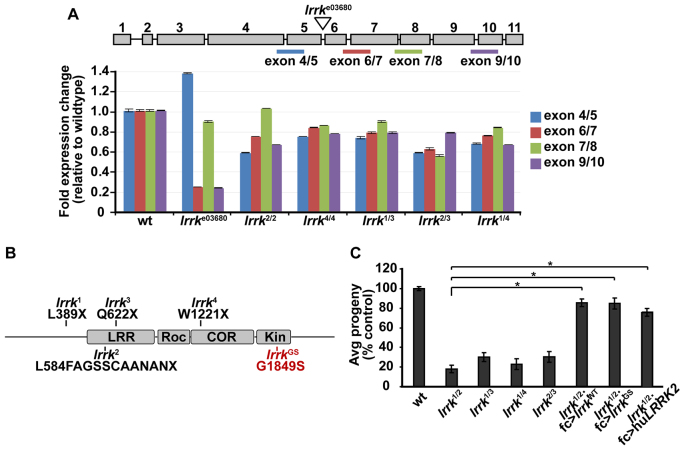
**Characterization of *lrrk* NS alleles, which result in reduced female fertility.** (A) Quantitative RT-PCR analysis of *lrrk* alleles, expressed as fold change in transcript abundance relative to wild type. Expected products from primer pairs used in this analysis are depicted in the schematic of the *lrrk* genomic region above (exons 1–11 depicted as gray rectangles and introns depicted as black lines). There is marked heterogeneity in transcript abundance in *lrrk*^e03680^ homozygous flies depending on which primer pairs were used, ranging from a transcript abundance of 25% relative to wild type using primers flanking the exon 6/7 and 9/10 splice sites, to a 36% increase in transcript abundance using primers flanking the exon 4/5 splice site. Note that the PiggyBac insertion site in the *lrrk*^e03680^ line is within the intron between exons 5 and 6. In contrast, regardless of which primer pairs were used, transcript abundance in *lrrk* NS flies was either not significantly changed relative to wild type, or mildly reduced to 60–80% of control. Note that trans-heterozygous combinations of *lrrk* NS alleles were used for some of this analysis because background lethal or semi-lethal mutations in the *lrrk*^1^ and *lrrk*^2^ stocks make obtaining adult homozygous flies difficult. (B) Schematic representation of Lrrk protein domains (LRR, leucine-rich repeats; Roc, GTPase domain; Cor, C-terminal of Roc domain; Kin, kinase domain), showing locations of nonsense mutations in *lrrk* NS alleles (*lrrk*^1^–*lrrk*^4^). Note that the *lrrk*^2^ allele is generated by a single-nucleotide insertion that results in a frameshift that creates a new stop codon 11 residues downstream. Shown in red is the location of the G1849S mutation within the kinase domain (referred to as *lrrk*^GS^), which is analogous to the most common PD-associated mutation in human *LRRK2*, G2019S. (C) As measured by average number of adult progeny produced per single female of the indicated genotype, trans-heterozygous combinations of *lrrk* NS alleles show markedly reduced female fertility, which can be rescued by targeted follicle-cell-specific expression of wild-type *lrrk* (*lrrk*^WT^), *lrrk^GS^* or human *LRRK2* (hu*LRRK2*). *n*=20 single females per genotype. *P<0.0001.

The best-characterized loss-of-function mutation in *Drosophila lrrk*, *lrrk*^e03680^, results from a PiggyBac insertion in a *lrrk* intron (Fig. 1A) (Imai et al., 2008; Lee et al., 2007; Matta et al., 2012; Wang et al., 2008). Previously, we reported a reduction in *lrrk* transcript levels caused by the *lrrk*^e03680^ allele by quantitative RT-PCR (Dodson et al., 2012). However, in a more detailed analysis using multiple primer pairs distributed across the *lrrk* genomic region, we found heterogeneity in *lrrk* transcript abundance in *lrrk*^e03680^ flies (Fig. 1A). For example, primer pairs spanning the exon 6/7 and exon 9/10 splice junctions yielded a transcript abundance of about 25% of control in *lrrk*^e03680^ homozygous flies, whereas primer pairs spanning the exon 7/8 junction yielded an abundance of 90% of control, and primers spanning the exon 4/5 junction gave a 36% increase in transcript abundance (Fig. 1A). These results were highly reproducible across multiple replicates, and suggest the presence of multiple transcripts that are differentially affected by the PiggyBac insertion, and indeed that some of these transcripts might be upregulated in *lrrk*^e03680^ flies.

These results suggested the possibility that the *lrrk*^e03680^ allele does not represent a true null owing to differentially expressed alternative isoforms. To generate new *lrrk* nonsense alleles, we used TILLING, a high-throughput method by which point mutations in a gene of interest are identified in a population of chemically mutagenized flies (Till et al., 2003). This yielded four ethyl methanesulfonate (EMS)-induced nonsense mutations in *lrrk* (these alleles are hereafter collectively referred to as *lrrk* NS). The truncated proteins encoded by all four of the *lrrk* NS alleles are predicted to lack the kinase domain, and three of the four would additionally lack the GTPase domain (Fig. 1B). In contrast to the *lrrk*^e03680^ allele, the *lrrk* NS alleles consistently and reproducibly yielded transcript abundances that were either not significantly changed compared with wild type, or that were modestly reduced to 60–80% of control (Fig. 1A), suggesting that the transcript abundance of the different *lrrk* isoforms is less differentially affected by the NS alleles than by the *lrrk*^e03680^ PiggyBac insertion. Unfortunately, using the only published antibody to *Drosophila* Lrrk (Imai et al., 2008), we were unable to detect a specific Lrrk signal in wild-type flies, nor in *lrrk* overexpression flies, under multiple experimental conditions. Thus, we are unable to assess the net effect of these alleles on Lrrk protein abundance.

Because alleles generated by EMS mutagenesis are likely to also carry background mutations, we exclusively focused our studies on trans-heterozygous combinations of these alleles because they would be highly unlikely to carry similar EMS-induced background mutations. All phenotypes described below were highly similar if not identical in all trans-heterozygous combinations of these four nonsense alleles. *lrrk* NS flies did not show any discernible defects in dopaminergic neuron number or morphology with age (supplementary material Fig. S1), which is consistent with what has previously been reported for *lrrk*^e03680^ flies (Wang et al., 2008). This is not unexpected given that dominant mutations in *LRRK2*, rather than recessive loss-of-function mutations, cause PD.

**Fig. 2.**
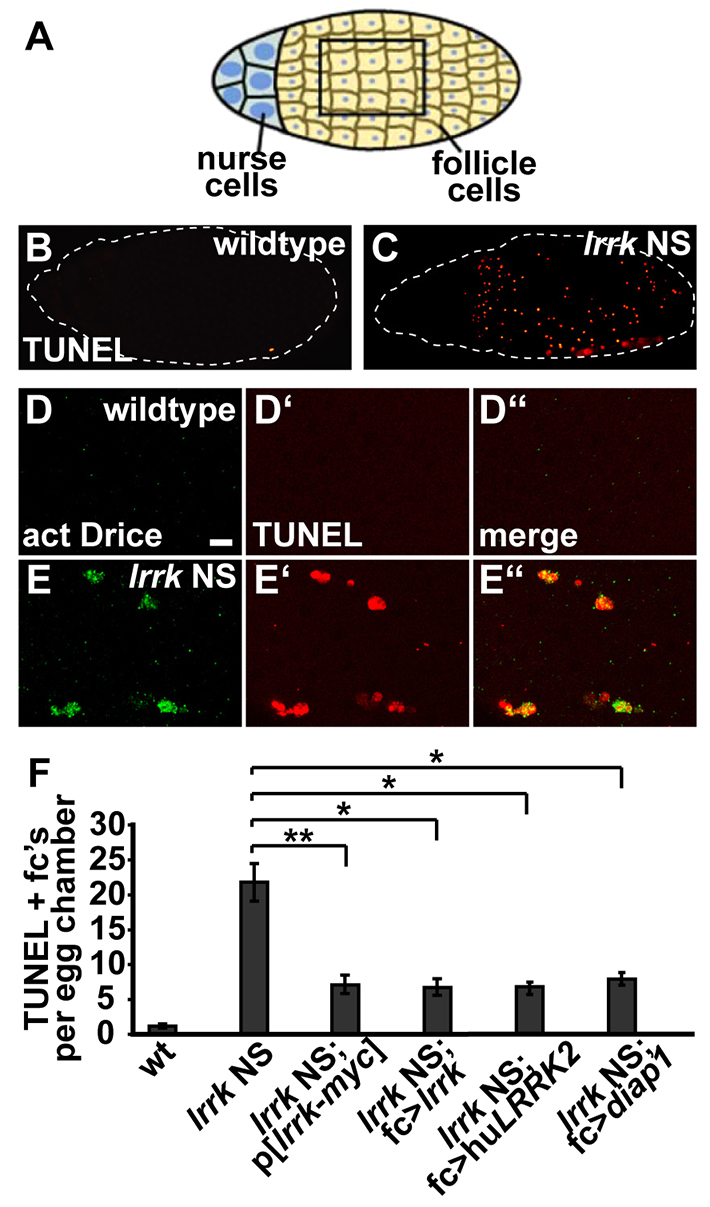
**Follicle cells undergo premature caspase-dependent cell death in *lrrk* NS flies.** (A) Schematic depicting a stage-12 egg chamber, with anterior to the left and posterior to the right. Most of the exterior of the egg chamber is composed of an epithelial layer of somatic follicle cells that surrounds the developing oocyte, whereas the anterior of the egg chamber contains the germline nurse cells, which are clonally related to the oocyte. (B–E) Note that, whereas B and C depict whole egg chambers, D and E are higher-magnification views of follicle cells, similar to what is outlined by the box in A. (B,C) Single stage-12 egg chambers from wild-type (B) and *lrrk*^1/4^ (C) females labeled by TUNEL to highlight apoptotic nuclei, show a dramatic increase in follicle cell death in *lrrk* NS egg chambers. The egg chamber is outlined with a dashed line for ease of visualization. Note that the area to the anterior (right) of the *lrrk* NS egg chamber (C) is devoid of TUNEL-positive signal because this area is occupied predominantly by nurse cells rather than follicle cells. (D,E) Higher-magnification images of follicle cells co-stained for activated caspase-3 (called Drice in *Drosophila*) (D,E) and TUNEL (D′,E′) demonstrates increased caspase activation in apoptotic follicle cells from stage-12 *lrrk*^1/2^ egg chambers (E) relative to wild type (D). Merged images show that, in *lrrk* NS flies, follicle cells that accumulate activated Drice are also TUNEL-positive (E″), suggesting that caspase activation occurs specifically in cells undergoing apoptosis. (F) Quantification of average number of TUNEL-positive follicle cells per egg chamber of the indicated genotype. Whereas *lrrk* NS alleles show a dramatic increase in follicle cell apoptosis relative to wild type, this is significantly rescued by a myc-tagged *lrrk* genomic rescue transgene, and by follicle-cell-specific expression of either *Drosophila lrrk* or human *LRRK2*. Apoptosis of *lrrk* NS follicle cells is also suppressed by expression of the caspase inhibitor *diap1*, suggesting that this process is caspase-dependent. *n*=56, 41, 58, 26, 107 and 61 egg chambers each, respectively, for the genotypes in panel F. *P<0.0001. **P=0.002. Scale bar in D: 10 μm.

Previously, we and others have reported that *lrrk*^e03680^ homozygous females show a dramatic reduction in female fertility (Dodson et al., 2012; Imai et al., 2008; Lee et al., 2007), which can be rescued by restoring wild-type *lrrk* function specifically in follicle cells (Dodson et al., 2012). Follicle cells are a somatic epithelial cell monolayer that surrounds the developing oocyte during oogenesis (Fig. 2A) (King, 1970; Spradling, 1993). Follicle cells have a number of advantages that make them an attractive cell biological system, including their large size, ease of accessibility and squamous morphology that aids in visualization of subcellular structures. As expected, all trans-heterozygous combinations of *lrrk* NS alleles showed reduced female fertility that could be rescued by follicle-cell-specific expression of wild-type *lrrk* (Fig. 1C). Expression of *lrrk*^GS^, which is analogous to the most common PD-causing mutation in human *LRRK2* (G2019S) (Bonifati, 2007; Taylor et al., 2006), also suppressed female infertility in *lrrk* NS flies (Fig. 1C). This suggests that *lrrk*^GS^ retains at least some of the functions of wild-type *lrrk*, consistent with what we have previously reported (Dodson et al., 2012). Moreover, follicle-cell-specific expression of human *LRRK2* also restored fertility to *lrrk* NS flies (Fig. 1C), suggesting that the human and *Drosophila* proteins are functionally conserved.

Egg chamber development in *Drosophila* is divided into 14 stages, and follicle cells normally undergo programmed cell death at the culmination of egg chamber development (King, 1970; Spradling, 1993). In *lrrk* NS flies, however, TUNEL staining revealed premature follicle cell death at stages 11–13 (Fig. 2C vs 2B; Fig. 2F). As with all other *lrrk* NS follicle cell phenotypes reported below, there was marked heterogeneity in the number of TUNEL-positive follicle cells per egg chamber, with some *lrrk* NS egg chambers severely affected and others appearing completely normal. Thus, quantification has been employed for *lrrk* NS follicle cell phenotypes where possible, such that the magnitude of the phenotype is averaged over all egg chambers. Premature follicle cell death in *lrrk* NS flies was rescued by targeted expression of *lrrk* or human *LRRK2* in follicle cells, and by a single copy of a *lrrk* genomic rescue transgene (Dodson et al., 2012) (Fig. 2F). *lrrk*^e03680^ homozygous flies did not show premature follicle cell death (supplementary material Fig. S2C), which is consistent with our hypothesis that this allele might not uncover the full range of *lrrk* loss-of-function phenotypes. Premature follicle cell death in *lrrk* NS flies was associated with increased staining for activated caspase-3 (called Drice in *Drosophila*) (Fig. 2E vs 2D), and double labeling with activated caspase-3 and TUNEL confirmed that caspase activation was specific to dying cells (Fig. 2E″ vs 2D″). Moreover, follicle cell death in *lrrk* NS flies was suppressed by expression of the caspase inhibitor *Drosophila inhibitor of apoptosis 1* (*diap-1*) (Hay et al., 1995) (Fig. 2F). Collectively, these data suggest that premature follicle cell death in *lrrk* NS flies is caspase-dependent.

### *lrrk* NS flies show massively expanded lysosomal compartments that aberrantly accumulate lipid and autophagic components

**Fig. 3.**
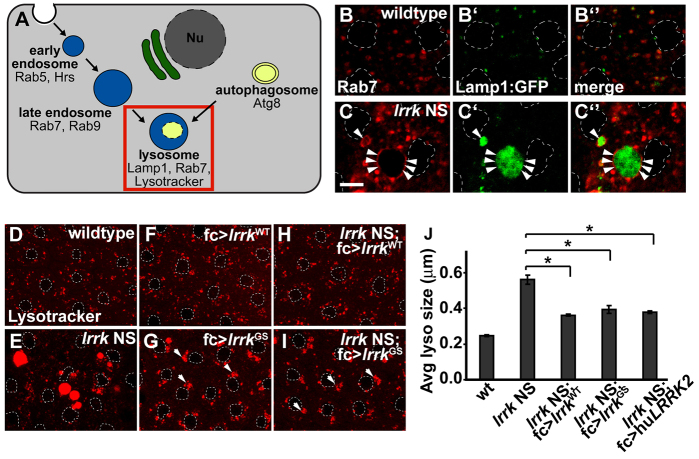
**Abnormally expanded late endosomal and lysosomal compartments in *lrrk* NS flies.** (A) Schematic depicting the endosomal and autophagy pathways, and highlighting markers for different pathway compartments. The lysosomal compartment, indicated by the red box, is the focus of this figure. Nu, nucleus. (B,C) Antibody staining for the late endosomal protein Rab7 in follicle cells from stage-12 wild-type (B) and *lrrk* NS (C) egg chambers shows dramatically enlarged Rab7-positive compartments in *lrrk* NS. These enlarged Rab7-positive compartments accumulate the lysosomal marker Lamp1:GFP (C′ vs B′). Arrowheads indicate Rab7 staining of the late endosomal membrane. (D–I) Staining of lysosomes with the acidophilic dye Lysotracker in the indicated genotypes shows dramatically enlarged lysosomes in *lrrk* NS follicle cells (E) relative to wild type (D); this phenotype can be rescued by follicle-cell-specific expression of wild-type *lrrk* (H). In an otherwise wild-type background, expression of *lrrk^GS^* (G), but not wild-type *lrrk* (F), results in perinuclear clustering of lysosomes. In *lrrk* NS flies, expression of *lrrk^GS^* suppresses the enlarged lysosome phenotype; however, perinuclear clustering of lysosomes is still observed (I). Arrowheads indicate perinuclear lysosome clusters. (J) Quantification of average lysosome size in the indicated genotypes as determined by Lysotracker staining of follicle cells from stage-12 egg chambers. Whereas *lrrk* NS results in a dramatic increase in average lysosome size, this phenotype is significantly suppressed by follicle-cell-specific expression of wild-type *lrrk*, *lrrk^GS^* or human *LRRK2*. *n*=16, 34, 16, 16 and 17 egg chambers each, respectively, for the genotypes in J. *P<0.0001. *lrrk* NS is *lrrk*^1/2^. Follicle cell nuclei are outlined by dashed white lines in B–I. Scale bar: 5 μm.

As we have previously reported, Lrrk localizes predominantly to the membranes of late endosomes and lysosomes, and to a lesser extent to early endosomes, *in vivo* (Dodson et al., 2012). We thus first asked whether premature follicle cell death in *lrrk* NS flies is associated with defects in the late endosomal and lysosomal compartments. Using multiple late endosomal and lysosomal markers, including Rab7 (Fig. 3C vs 3B), Lamp1:GFP (Fig. 3C′ vs 3B′) and the acidophilic dye Lysotracker (Fig. 3E vs 3D), we found that many *lrrk* NS follicle cells showed markedly enlarged late endosomal/lysosomal structures. There did not seem to be any appreciable alteration in lysosome position in *lrrk* NS flies: the enlarged lysosomes occurred in both the perinuclear region as well as the cell periphery. Expression of the caspase inhibitor *diap1* had no significant effect on lysosome enlargement in *lrrk* NS flies (supplementary material Fig. S3), suggesting that lysosome enlargement is not a consequence of caspase activation. In contrast to the massively enlarged lysosomal structures observed in *lrrk* NS flies, *lrrk*^e03680^ homozygous flies showed a mild increase in the size of the Rab7-positive late endosomal compartment in a subset of cells (supplementary material Fig. S2F) and no discernible change in lysosome morphology by Lysotracker staining (supplementary material Fig. S2I) (Dodson et al., 2012). Interestingly, the massive accumulation of Lamp1:GFP within enlarged lysosomes in *lrrk* NS flies (Fig. 3C′) suggests that their expansion in size is associated with, and perhaps due to, a defect in the degradative properties of the lysosome, because Lamp1:GFP is normally degraded rapidly upon reaching the lysosome (Pulipparacharuvil et al., 2005).

The enlarged-lysosome phenotype of *lrrk* NS flies could be rescued by follicle-cell-specific expression of either *Drosophila lrrk* (Fig. 3H vs 3E; Fig. 3J) or human *LRRK2* (Fig. 3J). Previously, we reported that the pathogenic Lrrk^GS^ (G2019S) mutation alters the function of Lrrk such that Lrrk^GS^ promotes perinuclear lysosome transport, whereas equivalent expression of Lrrk^WT^ (wild type) inhibits this process (Dodson et al., 2012). Interestingly, expression of *lrrk*^GS^ suppressed the enlarged-lysosome phenotype caused by *lrrk* NS as efficiently as did expression of *lrrk*^WT^ (Fig. 3E–J). The finding that the suppression of lysosome enlargement in *lrrk* NS flies was identical between *lrrk*^GS^ and *lrrk*^WT^ indicates that Lrrk^GS^ retains at least some of the functions of Lrrk^WT^, at least with respect to the Lrrk-dependent processes controlling lysosome size. However, perinuclear lysosome clustering was still observed with expression of *lrrk*^GS^ (Fig. 3G,I), but not *lrrk*^WT^ (Fig. 3F,H), regardless of whether these were expressed in the wild-type or *lrrk* NS background. This is consistent with a model we previously proposed in which Lrrk^GS^ retains at least some of the normal functions of the wild-type protein, while also possessing neomorphic effects (Dodson et al., 2012).

**Fig. 4.**
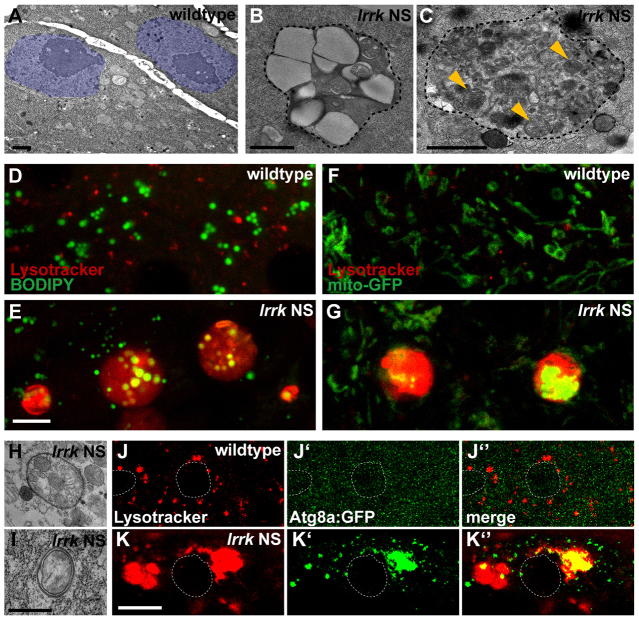
***lrrk* NS flies accumulate cytosolic material, including undigested organelles and lipid inclusions, within lysosomes, and accumulate autophagosomes.** (A–C) Transmission electron micrographs (TEMs) from wild-type (A) and *lrrk* NS mutant (B,C) follicle cells from stage-12 egg chambers. In wild-type follicle cells (A), no enlarged lysosomes are observed (follicle cell nuclei are highlighted in purple). Observed in the cytosol of *lrrk* NS flies (B,C) are aberrantly enlarged membrane-bound structures (outlined with dashed black line) filled with clear inclusions of lipid (B) and undigested cytosolic contents (C), including intact mitochondria (marked with yellow arrowheads). (D,E) Co-staining with Lysotracker (red) and the lipophilic dye BODIPY 493/503 (green) shows that the aberrantly enlarged lysosomes in follicle cells from *lrrk* NS flies (E) accumulate lipid inclusions, whereas this was not observed in wild type (D). (F,G) Similarly, the enlarged lysosomes in *lrrk* NS flies (G) also accumulate numerous mitochondria as labeled with a mitochondrially targeted GFP, whereas mitochondria always appeared to be distinct from lysosomes in wild-type follicle cells (F). (H,I) TEM images from *lrrk* NS follicle cells demonstrate the presence of double-membrane autophagosomes often containing intact mitochondria. Compare with wild-type follicle cells in A, in which autophagosomes are not observed by TEM analysis. (J,K) Lysotracker staining in follicle cells expressing the autophagosome marker Atg8a:GFP (J′,K′) in wild-type (J) and *lrrk* NS (K) follicle cells. In wild-type cells, Atg8a:GFP is mostly uniformly distributed throughout the cytosol (J′), indicating the relative absence of autophagosomes. In *lrrk* NS follicle cells, however, Atg8a:GFP shifts to a punctate distribution (K′), and these puncta occasionally, but not always, colocalize with Lysotracker (K″), indicating the presence of both autophagosomes and autolysosomes in *lrrk* NS flies. *lrrk* NS is *lrrk*^1/2^. Follicle cell nuclei are outlined by dashed white lines in J–K″. Scale bars: 1 μm in A–C and H,I, and 5 μm in D–G and J–K″.

On transmission electron microscopy (TEM) analysis, the expanded lysosomes in *lrrk* NS flies contained prominent large clear inclusions reminiscent of lipid (Fig. 4B), as well as undigested cytosolic contents, including intact mitochondria (Fig. 4C). These findings were confirmed with immunofluorescence analysis, in which the enlarged Lysotracker-positive lysosomes in *lrrk* NS flies also contained lipid, as labeled by the lipophilic dye BODIPY 493/503 (Fig. 4E), as well as mitochondria, as labeled by GFP fused to a mitochondrial targeting sequence (mitoGFP) (Fig. 4G). BODIPY 493/503 (Fig. 4D)- or mitoGFP (Fig. 4F)-positive signals were never seen within Lysotracker-positive lysosomes in stage-matched wild-type follicle cells. Thus, the aberrantly enlarged lysosomes in *lrrk* NS cells accumulate both lipid and intact mitochondria, suggesting a primary defect in the degradative properties of lysosomes in *lrrk* NS flies.

### Autophagy is dysregulated in *lrrk* NS flies

The lysosome is the final degradative compartment for multiple cellular pathways, including both the endocytic and autophagy pathways. The finding of intact organelles within enlarged lysosomes in *lrrk* NS flies suggests that at least some of these lysosomal contents are derived from the autophagy pathway. The hallmark of autophagy is the formation of double-membrane structures called autophagosomes, which label specifically with the marker Atg8a:GFP (Fig. 3A), the *Drosophila* homolog of mammalian LC3 (Geng and Klionsky, 2008). Autophagosomes form in the cytosol, engulf cytosolic components and are then transported to the lysosome for degradation (Feng et al., 2014). In *lrrk* NS follicle cells, autophagosomes were indeed apparent both by TEM analysis (Fig. 4H,I) and by a marked redistribution of Atg8a:GFP from a diffuse pattern in wild-type cells (Fig. 4J′), indicating a relative absence of autophagosomes, to predominantly punctate pattern in *lrrk* NS cells (Fig. 4K′). Moreover, autophagosomes were not observed in stage-matched wild-type follicle cells by TEM analysis (Fig. 4A). These puncta of Atg8a:GFP in *lrrk* NS flies often localized to Lysotracker-positive lysosomes, but were also frequently distinct from lysosomes (Fig. 4K″). Thus, *lrrk* NS cells show an increase in structures derived from autophagy; this increase could be due to either increased autophagy induction, decreased clearance of autophagosomes and autolysosomes, or both.

**Fig. 5.**
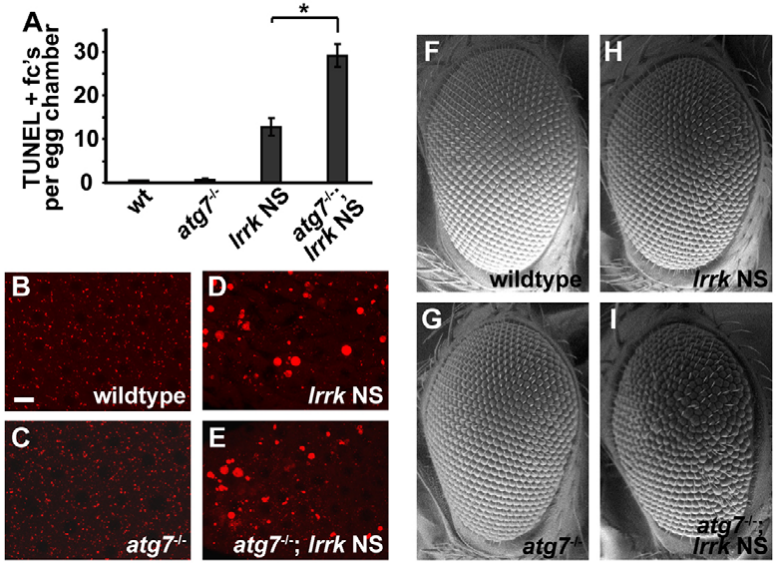
***atg7* loss-of-function enhances cell death in *lrrk* NS flies, but does not enhance lysosome enlargement.** (A) Average number of TUNEL-positive follicle cell nuclei in the indicated genotypes shows an increase in follicle cell death in *atg7*/*lrrk* NS double mutants relative to *lrrk* NS alone. *atg7* loss-of-function alone did not cause any significant follicle cell death by TUNEL staining. *n*=61, 63, 62 and 57 egg chambers each, respectively, for the genotypes in A. *P<0.0001. (B–E) Lysotracker staining of the indicated genotypes shows no significant enhancement of lysosome enlargement in *atg7*/*lrrk* NS double mutants (E) versus *lrrk* NS mutants alone (D). Relative to wild type (B), *atg7* loss-of-function alone (C) has no significant effect on lysosome morphology. (F–I) Scanning electron micrographs of adult fly eyes of the indicated genotypes shows that *atg7*/*lrrk* NS double mutants display a mild rough-eye phenotype (I). In contrast, eye morphology is indistinguishable from wild type (F) in *atg7* (G) and *lrrk* NS (H) single mutants. *lrrk* NS is *lrrk*^1/1^. Scale bar: 5 μm for B–E.

To evaluate this further, we studied the consequences of suppressing autophagy in *lrrk* NS flies. Atg7 is a component of the ubiquitin-like conjugation system required for autophagosome formation (Geng and Klionsky, 2008), and removing the function of *atg7* in *Drosophila* has been shown to inhibit starvation-induced autophagy in the fat body (Juhász et al., 2007). Interestingly, we found that removing *atg7* function in *lrrk* NS flies resulted in a significant increase in follicle cell death as assessed by TUNEL staining (Fig. 5A). This was not associated with any significant enhancement in the lysosome-enlargement phenotype observed in *lrrk* NS flies (Fig. 5B–E). These *atg7*/*lrrk* NS double mutants also displayed a mild rough-eye phenotype (Fig. 5I), which was not observed in either single mutant alone (Fig. 5G,H). The increased toxicity observed in *atg7*/*lrrk* double mutants versus *lrrk* NS flies alone is consistent with autophagy induction as a prosurvival response in *lrrk* NS flies. That this increased toxicity in the double mutants is not associated with enhancement of the *lrrk* NS lysosome-enlargement defect suggests that the lysosomal defects in *lrrk* NS flies are independent of the autophagy pathway.

### *lrrk* NS flies display aberrant early endosomes laden with ubiquitylated cargoes

**Fig. 6.**
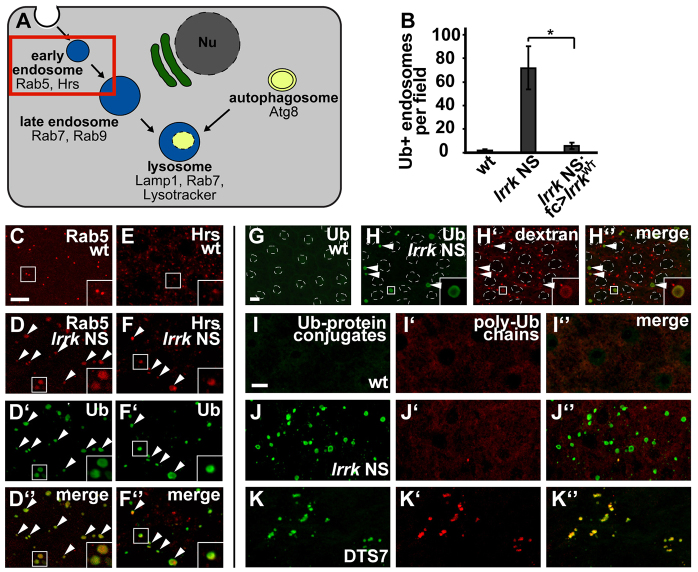
***lrrk* NS follicle cells accumulate enlarged early endosomes laden with ubiquitylated cargo proteins.** (A) Schematic depicting the endosomal and autophagy pathways. Highlighted is the early endosome compartment, which is specifically labeled with anti-Rab5 and anti-Hrs antibodies. Nu, nucleus. (B) Quantification of the number of ubiquitin-positive endosomes per microscope field in the indicated genotypes, showing a dramatic increase in the number of endosomes laden with ubiquitylated proteins in *lrrk* NS flies, which is significantly suppressed by follicle-cell-specific expression of wild-type *lrrk*. *n*=18, 15 and 20 egg chambers each, respectively, for the genotypes in B. *P=0.02. (C–F) Antibody staining for the early endosomal proteins Rab5 (C,D) and Hrs (E,F) in wild-type (C,E) and *lrrk* NS (D,F) stage-12 follicle cells. In *lrrk* NS cells, the Rab5 (D vs C)- and Hrs (F vs E)-positive compartments are dramatically expanded. Co-staining with an antibody (D′ and F′) that recognizes either monoubiquitin or polyubiquitin chains conjugated to any target protein, but not free ubiquitin, in *lrrk* NS cells shows that these enlarged Rab5 (D″)- and Hrs (F″)-positive compartments are laden with ubiquitylated proteins. Arrowheads indicate enlarged endosomes labeled by Rab5/Hrs and ubiquitin. (G,H) In wild-type follicle cells (G), there is no significant labeling by the antibody recognizing ubiquitin-protein conjugates, whereas, in *lrrk* NS follicle cells (H), these large ubiquitin-positive structures are frequently seen. Note that the ubiquitin-protein conjugate staining in *lrrk* NS cells frequently appears as a halo surrounding a non-stained central region (inset), suggesting accumulation of ubiquitylated proteins on endosomal membranes. This is consistent with the co-labeling of these structures with the early endosomal proteins Rab5 and Hrs as seen in D and F. These accumulations of ubiquitin-protein conjugates in *lrrk* NS also colocalize with endocytosed dextran (H′ and H″), confirming the identity of these structures as endosomes. Arrowheads indicate enlarged endosomes labeled by dextran and ubiquitin. (I–K) The ubiquitin-protein conjugates that accumulate in *lrrk* NS flies are predominantly monoubiquitylated. In wild-type follicle cells (I), there is no significant staining with an antibody recognizing ubiquitin-protein conjugates (I), nor with an antibody recognizing polyubiquitin chains (I′). In *lrrk* NS cells, the accumulations of ubiquitin-protein conjugates (J) do not co-label with the antibody to polyubiquitin chains (J′,J″), suggesting that these accumulations are predominantly monoubiquitylated proteins. As a positive control, the dominant-negative proteasome subunit DTS7 accumulates inclusions that stain positive both for ubiquitin-protein conjugates (K) and for polyubiquitin chains (K′,K″). *lrrk* NS is *lrrk*^1/2^. Follicle cell nuclei are outlined by dashed white lines in G and H. Scale bars: 5 μm in all images.

Previously, we reported that Lrrk also physically binds to the early endosomal protein Rab5 (Dodson et al., 2012), which plays a vital role in the maturation of early endosomes to late endosomes and, thus, in endosomal substrate delivery to the lysosome (Huotari and Helenius, 2011). Genetic and physical interactions have also been reported between mammalian LRRK2 and Rab5 in cell-culture models under conditions of *LRRK2* overexpression (Heo et al., 2010; Shin et al., 2008). Thus, we investigated whether *lrrk* also acts to regulate cargo delivery to the lysosome in the endocytic pathway. Interestingly, we found that the early endosomes, marked by either anti-Rab5 (Fig. 6D vs 6C) or anti-hepatocyte-growth-factor-regulated tyrosine kinase substrate (Hrs) antibodies (Fig. 6F vs 6E), were enlarged in *lrrk* NS follicle cells relative to wild type. In the endocytic pathway, monoubiquitylation serves as a sorting signal for protein cargoes that are bound for the lysosome to be degraded (Piper and Luzio, 2007; Raiborg and Stenmark, 2009). Interestingly, *lrrk* NS cells showed a marked accumulation of ubiquitylated proteins (as identified by an antibody that recognizes mono- or polyubiquitin conjugated to any target protein, but not free ubiquitin) (Fig. 6H vs 6G; Fig. 6B). Interestingly, this ubiquitin-positive staining often appeared as halos surrounding a non-stained central region (Fig. 6H, inset), suggesting the accumulation of ubiquitylated cargoes on vesicle membranes. Consistent with this hypothesis, the ubiquitylated-protein accumulations in *lrrk* NS cells colocalized with the expanded anti-Rab5-positive (Fig. 6D″) and anti-Hrs-positive (Fig. 6F″) early endosomes. These structures also accumulated the endocytic tracer dextran (Fig. 6H″), which is taken up from the extracellular space via endocytosis and subsequently trafficked through the endocytic pathway, thus confirming the identity of these structures as endosomes. Moreover, these ubiquitin accumulations consisted primarily of monoubiquitylated proteins, as evidenced by a lack of staining with an antibody that recognizes polyubiquitin chains (Fig. 6J′). This is distinct from what is observed with expression of the dominant-negative proteosome subunit DTS7 (serving as a control), which resulted in the accumulation of predominantly polyubiquitylated proteins recognized by both antibodies (Fig. 6K″). Importantly, the accumulation of monoubiquitylated proteins in *lrrk* NS flies could be rescued by follicle-cell-specific expression of wild-type *lrrk* (Fig. 6B). *lrrk*^e03680^ homozygous flies showed a similar, albeit weaker, accumulation of ubiquitylated proteins (supplementary material Fig. S4C versus S4B), which also colocalized with internalized dextran (supplementary material Fig. S4C″) and Hrs (supplementary material Fig. S4F″). Collectively, these results show that *lrrk* NS flies accumulate aberrantly enlarged early endosomes that are laden with monoubiquitylated endosomal cargo proteins, suggesting a defect in substrate delivery to the lysosome in the endocytic pathway.

### *lrrk* NS phenotypes are rescued by expression of constitutively active *rab9*

Previously, we found, from predominantly overexpression-based studies, that *lrrk* inhibits *rab7*-dependent perinuclear lysosome clustering (Dodson et al., 2012). We therefore asked whether the lysosomal-enlargement phenotype seen with *lrrk* loss-of-function is due to an interaction between *lrrk* and *rab7*. However, we found that *lrrk* loss-of-function does not phenocopy the lysosome clustering caused by expression of a constitutively active form of *rab7* (*rab7*^CA^) (supplementary material Fig. S5B), as would be expected according to this model. Moreover, *rab7*^CA^ had no effect on lysosome enlargement in *lrrk* NS flies (supplementary material Fig. S5E vs S5D,G). In addition, average lysosome size was no different in *lrrk* NS flies alone compared with *lrrk* NS flies expressing a dominant-negative version of *rab7* (*rab7*^DN^) (supplementary material Fig. S5G). These *lrrk* NS/*rab7*^DN^ flies did not show the massively enlarged lysosomes often seen in *lrrk* NS flies (supplementary material Fig. S5F vs S5D), but rather showed a more moderate but consistent lysosome-enlargement phenotype, the net result being no overall change in average lysosome size compared with *lrrk* NS (supplementary material Fig. S5G). This result is not unexpected given that dominant-negative *rab7* results in lysosome dispersal and decreased lysosome fusion (Bucci et al., 2000). Taken together, these data suggest that the lysosome enlargement observed in *lrrk* NS flies is at least partially Rab7-independent.

Recently, it was reported that LRRK2 forms a complex with VPS35 (MacLeod et al., 2013), which is a core component of the retromer complex, involved in retrograde trafficking of cargoes from the endocytic pathway to the trans-Golgi network (Bonifacino and Rojas, 2006). This pathway is required for the retrograde transport of multiple substrates, including the mannose 6-phosphate receptor (MPR) (Arighi et al., 2004; Hierro et al., 2007; Seaman, 2004), a process that also requires the late endosomal GTPase Rab9 (Barbero et al., 2002; Lombardi et al., 1993; Riederer et al., 1994). Overexpression of *LRRK2* G2019S (Lrrk^GS^) has been reported to disrupt the normal trafficking of the MPR; this disruption can be rescued by overexpression of *Vps35* (MacLeod et al., 2013). Inhibition of the retromer pathway increases lysosomal degradation of the MPR and thereby results in swelling of lysosomes due to intra-lysosomal accumulation of undigested material, presumably due to defects in trafficking of hydrolases to the lysosome (Arighi et al., 2004). Because this phenotype is highly reminiscent of what we have observed in *lrrk* NS flies, we hypothesized that augmenting retrograde transport might suppress *lrrk* NS phenotypes.

**Fig. 7.**
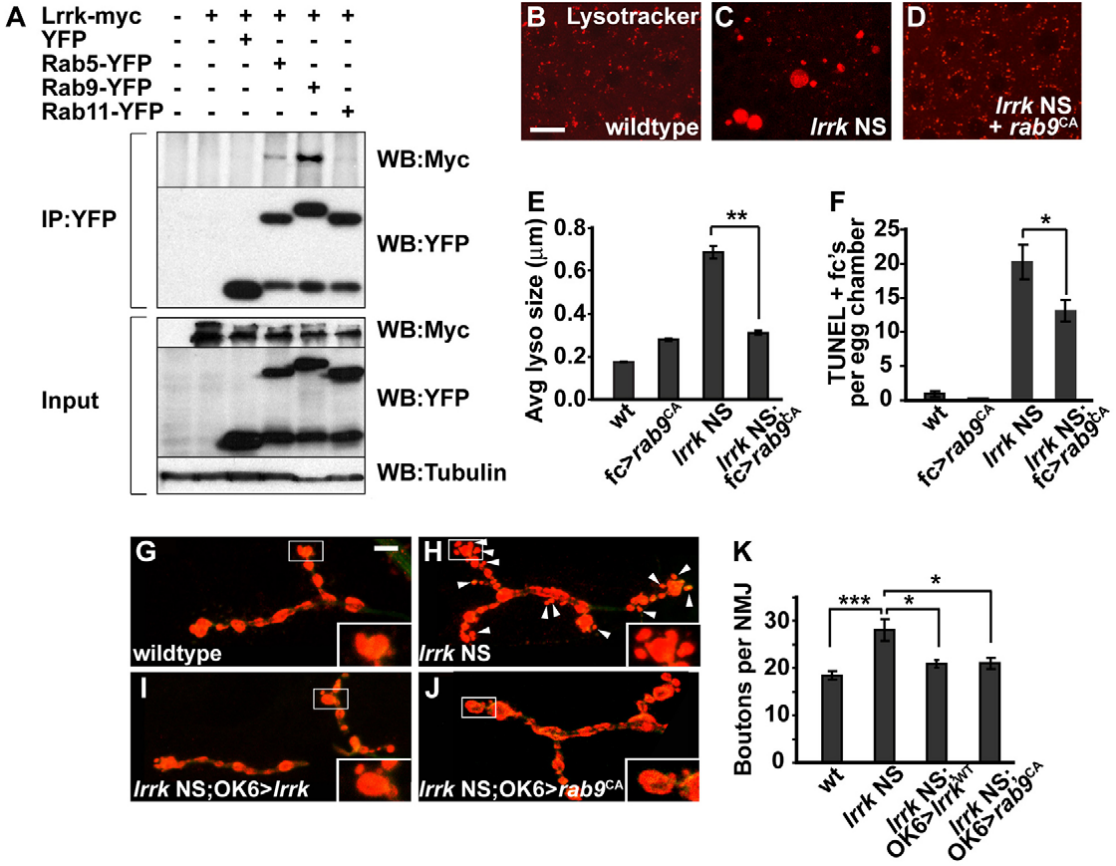
**Lrrk physically binds to Rab9, and expression of activated *rab9* rescues *lrrk* NS phenotypes.** (A) In lysates from transiently transfected cultured *Drosophila* S2 cells, Lrrk-Myc co-immunoprecipitates with YFP-tagged Rab5 and more strongly with Rab9, but not with Rab11 or YFP alone. (B–D) Lysotracker staining of stage-12 follicle cells from wild-type flies (B), *lrrk* NS flies (C) and *lrrk* NS flies expressing constitutively active *rab9* (*rab9*^CA^) specifically in follicle cells (D) shows dramatic suppression of the abnormal lysosome morphology seen in *lrrk* NS flies when *rab9* is expressed. (E) Quantification of average lysosome size in Lysotracker-stained follicle cells shows suppression of lysosome enlargement by *rab9*^CA^ in *lrrk* NS flies. *n*=16, 16, 34 and 33 egg chambers each, respectively, for the genotypes in E. **P<0.0001. (F) Similarly, premature follicle cell death in *lrrk* NS flies, as assessed by TUNEL staining, is suppressed by *rab9*^CA^. *n*=61, 68, 99 and 102 egg chambers each, respectively, for the genotypes in F. *P<0.05. (G–J) Staining of the presynaptic motor neuron at the neuromuscular junction (NMJ) at abdominal segment 4, muscle 4 in late third instar larvae with anti-cysteine string protein (CSP; red), which labels synaptic vesicles. Compared with wild type (G), *lrrk* NS larvae (H) show an overgrowth phenotype in which numerous small satellite boutons (marked with arrowheads) form adjacent to the larger normal boutons. This overgrowth phenotype is significantly suppressed by expression of either wild-type *lrrk* (I) or *rab9*^CA^ (J) using the motor neuron-specific OK6-Gal4 driver. (K) Quantification of the numbers of boutons per NMJ in the indicated genotypes shows significant suppression of *lrrk* NS synaptic overgrowth with expression of both wild-type *lrrk* and *rab9*^CA^. *n*=27 NMJs analyzed for each genotype in K. ***P=0.0003, *P<0.05. *lrrk* NS is *lrrk*^1/2^. Scale bars: 5 μm in B–D, 10 μm in G–J.

As we have previously reported, *Drosophila* Lrrk colocalizes with Rab9 at the late-endosome membrane (Dodson et al., 2012). Interestingly, we found that Lrrk-myc co-immunoprecipitated with Rab9-YFP, but did not show significant binding with either YFP alone or with Rab11-YFP (Fig. 7A), which served as controls. Expression of a constitutively active form of *rab9* (*rab9*^CA^) in follicle cells resulted in marked suppression of the lysosome-enlargement phenotype observed in *lrrk* NS cells (Fig. 7D vs 7C; Fig. 7E). Similarly, *rab9*^CA^ significantly, although incompletely, suppressed premature follicle cell death in *lrrk* NS cells (Fig. 7F). One mechanism by which *lrrk* could be required for *rab9*-dependent processes is through regulation of the membrane recruitment of Rab9; however, we did not detect any significant difference in the subcellular localization of Rab9 between the wild-type and *lrrk*-NS-mutant backgrounds (supplementary material Fig. S6). Collectively, these data suggest that augmenting Rab9 function can, at least in part, bypass the need for *lrrk*.

To further substantiate the finding that overactivation of *rab9* can bypass the requirement for *lrrk*, we examined genetic interactions between *lrrk* and *rab9* in another tissue that *lrrk* has been shown to act on: the larval NMJ. Previously, it has been reported that *lrrk*^e03680^ mutants show a defect in NMJ morphology characterized by overgrowth of the synapse between the presynaptic motor neuron and postsynaptic muscle cell, with increased branching and number of boutons (Lee et al., 2010). We observed a similar, albeit stronger, phenotype in *lrrk* NS flies, where the synaptic overgrowth was mostly observed as small supernumerary satellite boutons surrounding the larger normal boutons (Fig. 7H vs 7G; Fig. 7K). This phenotype could be rescued by motor-neuron-specific expression of wild-type *lrrk* (Fig. 7I vs 7H; Fig. 7K). As observed in follicle cells, expression of *rab9*^CA^ significantly suppressed the NMJ morphology defect in *lrrk* NS flies (Fig. 7J vs 7H; Fig. 7K). Together, these results suggest that *lrrk* plays a role in retromer-mediated endosomal recycling, and that augmentation of late endosome-to-Golgi retrograde transport can bypass the requirement for *lrrk*.

## DISCUSSION

Here, we provide evidence of a vital physiological role for a *LRRK2* homolog in endolysosomal membrane transport and autophagy *in vivo*, by characterizing novel loss-of-function nonsense alleles in *Drosophila lrrk*. These *lrrk* NS alleles cause accumulation of markedly enlarged lysosomes full of undigested lipid and other cytosolic contents, as well as accumulation of autophagosomes and enlarged early endosomes laden with monoubiquitylated cargo proteins. The interactions that we describe between *lrrk* and *rab9* suggest that the role of Lrrk in maintaining proper lysosome function involves the Rab9-dependent late-endosome-to-Golgi pathway.

Whereas the increase in perinuclear lysosome clustering caused by expression of *lrrk*^GS^ is *rab7*-dependent (Dodson et al., 2012), the effects of *lrrk* loss-of-function on lysosome size are *rab7*-independent, and rather seem to be mediated through the Rab9-retromer pathway. *rab9* promotes retromer-dependent recycling of cargoes from late endosomes to the Golgi (Barbero et al., 2002; Lombardi et al., 1993; Riederer et al., 1994). Defects in retromer-dependent cargo trafficking lead to impaired lysosomal degradation and the accumulation of undigested materials within lysosomes (Arighi et al., 2004), a phenotype highly reminiscent of what is seen in *lrrk* NS flies. Rab9 and Lrrk colocalize at the late endosomal membrane (Dodson et al., 2012), and, as we show in this work, physically bind to one another. Augmentation of *rab9* activity rescues lysosomal enlargement and premature follicle cell death in *lrrk* NS flies. Enhancing *rab9* activity also rescues the NMJ morphology defect in *lrrk* NS flies, which have an NMJ phenotype identical to that reported with loss-of-function of the retromer component *Vps35* (Korolchuk et al., 2007). Thus, in multiple tissues, augmentation of *rab9* activity bypasses the need for *lrrk*, suggesting a role for *lrrk* in the Rab9-retromer pathway.

Defects in the autophagy pathway have previously been reported in the context of *lrrk* loss-of-function, but the results have been conflicting, including the finding of decreased autophagosome formation with *LRRK2* knockdown in immune cells (Schapansky et al., 2014) and biphasic alterations in autophagy in the kidney of *LRRK2* knockout mice, with markers of autophagy increased at 7 months of age and reduced at 20 months (Tong et al., 2012). Here, we report increased autophagic structures in *lrrk* NS flies, including both autophagosomes and autolysosomes. Although there is probably a component of decreased turnover of autophagic structures due to the lysosome dysfunction that occurs in *lrrk* NS flies, our data also suggest that autophagy induction is increased in *lrrk* NS flies, possibly as a prosurvival stress response to lysosome dysfunction or ubiquitylated-protein accumulation. This hypothesis is supported by the increased toxicity in *lrrk* NS flies when *atg7* function is removed. Caspase-dependent apoptosis seems to be the end result when the autophagic response is insufficient, and occurs downstream of both lysosome dysfunction and autophagy induction. We cannot, however, exclude the possibility that *lrrk* loss-of-function increases autophagy activation via a more direct mechanism. Interestingly, *rab9* has been implicated in autophagosome formation in mammalian cells in an alternative *atg7*-independent autophagy pathway that can be induced by certain cellular stressors (Nishida et al., 2009). That some *lrrk* NS phenotypes are enhanced by *atg7* loss-of-function suggests that autophagy induction in the context of *lrrk* loss-of-function is *atg7*-dependent rather than *atg7*-independent. Still, it remains an intriguing possibility that *lrrk* could directly regulate autophagy induction, perhaps via its interaction with *rab9*.

Another important question is whether the defects seen in *lrrk* NS flies at both the early endosomal compartment and the late endosomal/lysosomal compartments reflect distinct roles for Lrrk at multiple endolysosomal transport steps. For example, via its physical binding to the early endosomal protein Rab5, which we and others have reported (Dodson et al., 2012; Shin et al., 2008), LRRK2/Lrrk might exert a distinct Rab9-independent role in the trafficking of early endosomes. Interestingly, loss-of-function of *Drosophila lrrk* has also been reported to impair synaptic vesicle endocytosis via an interaction with endophilin A, which is involved in the early membrane tubulation steps involved in vesicle budding from the plasma membrane (Matta et al., 2012). Thus, Lrrk might function as a general regulator of multiple distinct membrane transport steps.

Although our work has focused predominantly on follicle cells in the *Drosophila* ovary owing to the aforementioned benefits as a cell biological model system, we believe that our results have significance to the function of *lrrk* in neurons for several reasons. Lysosomal defects in *lrrk* NS flies can be rescued by human *LRRK2*, suggesting functional conservation of the endolysosomal functions of the two proteins. Moreover, both follicle cell lysosomal defects and abnormal NMJ morphology in *lrrk* NS flies are rescued by augmenting *rab9* function, suggesting similar defects in *rab9*-dependent membrane transport processes due to *lrrk* loss-of-function in both neuronal and non-neuronal tissues. Why might follicle cells be particularly sensitive to the effects of *lrrk* loss-of-function? Ultrastructural analyses have reported an increase in the number of lysosomes in follicle cells from stage-12 egg chambers (Cummings and King, 1970), the stage at which lysosome defects in *lrrk* NS flies become apparent. Moreover, this stage-specific increase in lysosome abundance plays a role in the degradation of nurse cells, which are germline cells clonally related to the oocyte that undergo programmed cell death at the culmination of oogenesis (King, 1970; Spradling, 1993). Thus, we speculate that an increased requirement for lysosomal degradation in late-stage follicle cells could underlie the sensitivity of these cells to *lrrk* loss-of-function.

Our work demonstrates a vital physiological role for endogenous *lrrk* in endolysosomal membrane transport and lysosome function *in vivo*, and suggests that the toxicity of the pathogenic mutant *lrrk*^GS^ allele is related to dysregulation of its endolysosomal functions. These findings have important implications for the development of inhibitor-based therapies for PD that target LRRK2, because excessive inhibition might result in endolysosomal dysfunction and thus cellular toxicity. Regulation of lysosomal function and autophagy have emerged as key themes in the pathogenesis of neurodegenerative diseases, from inherited diseases of lysosomal storage to diseases such as PD, involving the accumulation of protein inclusions. Together with a striking amount of recent genetic data linking PD risk to genes involved in endolysosomal functions, including β-glucocerebrosidase (Aharon-Peretz et al., 2004; Gan-Or et al., 2008), *ATP13A2* (Ramirez et al., 2006), *VPS35* (Vilariño-Güell et al., 2011; Zimprich et al., 2011) and *RAB7L1* (Gan-Or et al., 2012), our data establishes dysfunction of the endolysosomal pathway as a central pathogenic mechanism in PD.

## MATERIALS AND METHODS

### Molecular biology

The UAS-*lrrk*, UAS-*lrrk*^GS^ and CaSpeR-*lrrk* genomic rescue constructs were previously described (Dodson et al., 2012). For UAS-*LRRK2*, the human *LRRK2* cDNA was introduced into the pTHW gateway vector (which contains an N-terminal HA tag as well as the UASt promoter) by recombination. All cloned PCR products were confirmed by DNA sequencing. The Rab-YFP constructs were obtained from Matthew Scott (Stanford University, Palo Alto, CA).

### RNA purification and quantitative PCR

RNA was isolated from 1-day-old flies using Trizol, and purified using Phase Lock Gel Tubes to remove DNA. Two independent cDNA synthesis reactions were performed for each genotype using Clontech EcoDry Premix cDNA Kit (Double Primed Oligo-dT and Random Hexamers), after normalizing the amount of input RNA across genotypes. Quantitative PCR was performed in triplicate for each cDNA reaction and the six values for each genotype across the two cDNA reactions averaged. All primer pairs spanned an intron as depicted in Fig. 1A. Values were normalized to the geometric mean of three control genes, *rpl32*, *eIF1a* and *aTub84B*, for each genotype, and then normalized to wild type to calculate a fold change in gene expression.

### Drosophila genetics

To generate loss-of-function mutations in *lrrk*, we used TILLING (Till et al., 2003), which is a method for detecting point mutations in a gene of interest following chemical mutagenesis. EMS mutations were generated in a prior screen (Koundakjian et al., 2004), and were recovered using the Drosophila Tilling Service (Fred Hutchinson Cancer Research Center). *lrrk*^1^, *lrrk*^3^ and *lrrk*^4^ are nonsense alleles resulting from substitution of a stop codon for L389, Q622 and W1221, respectively. *lrrk*^2^, which was recovered from a mixed stock, has a single-nucleotide insertion changing L584 to F, and resulting in a frameshift that generates a stop codon 11 codons downstream. *lrrk*^e03680^ results from the insertion of a PiggyBac transposable element in the intron between exons 5 and 6, and was obtained from the Harvard Drosophila Stock Center. The follicle-cell-specific driver CY2-GAL4 was a gift from Celeste Berg (University of Washington, Seattle, WA). *atg7* mutants were obtained from Thomas Neufeld (Juhász et al., 2007). UAS-*rab7*^DN^, -*rab7*^CA^ and -*rab9*^CA^ are as described (Zhang et al., 2007). UASp-*diap1* flies were obtained from Bruce Hay (California Institute of Technology, Pasadena, CA) and Andreas Bergann (University of Massachusetts, Worchester, MA). All other stocks are from the Bloomington Drosophila Stock Center. For experiments involving transgenic flies, multiple lines were generated (Rainbow Transgenic Flies) and tested for each transgene. *Drosophila* strains were maintained in a 25°C humidified incubator unless otherwise noted.

### Immunofluorescence and confocal microscopy

For follicle cell experiments, freshly eclosed females were maintained on wet yeast paste for 24 hours prior to ovary dissection, and individual stage 11–13 egg chambers were hand dissected following fixation. The NMJ at abdominal segment 4, muscle 4 was used for all NMJ experiments. All tissues were fixed in either 3.7% formaldehyde or 4% paraformaldehyde in PBS. PBS + 0.1% Triton X-100 (or 0.2% Tween) + 2% BSA was used for blocking and antibody incubations. The following primary antibodies were used for immunocytochemistry: mouse anti-ubiquitin protein conjugates (Biomol, FK2, 1:300), mouse anti-polyubiquitin chains (Biomol, FK1, 1:25), rabbit anti-Rab5 (Abcam, 1:20), rabbit anti-Rab7 [a gift from Yashodhan Chinchore (Harvard University, Boston, MA) and Patrick Dolph (Dartmouth University, Hanover, NH), 1:100], mouse anti-tyrosine hydroxylase (Immunostar, 1:300), guinea pig anti-Hrs [a gift from Hugo Bellen (Baylor University, Houston, TX), 1:100], rabbit anti-cleaved-caspase-3 (Cell Signaling, 1:300), mouse anti-CSP (DHSB, 1:10) and fluorescein-conjugated anti-HRP (1:250). Secondary antibodies were Alexa Fluor 488 or 568 (Molecular Probes, 1:250). For Lysotracker staining, ovaries were dissected and incubated for 15 minutes in 100 nM Lysotracker red DND-99 (Molecular Probes), washed briefly, then mounted and imaged immediately, all in Schneider’s Drosophila Medium (Gibco). For lipid visualization, the Lysotracker staining solution was supplemented with 2 mg/ml BODIPY 493/503 (Molecular Probes). Images were obtained with a Zeiss LSM5 confocal microscope. All follicle cell images shown are from stage-12 or -13 egg chambers.

### Dextran uptake assay

Freshly dissected stage-12 egg chambers were incubated in 0.5 mM Texas-Red–dextran (3000 MW, Invitrogen) for 15 minutes, washed briefly five times, then incubated for 30 minutes prior to fixation in 4% paraformaldehyde. All steps were performed at 25°C in Schneider’s Drosophila Medium (Gibco).

### TUNEL assay

Ovaries were fixed in 3.7% formaldehyde for 20 minutes, washed in PBS, and stage 11–13 egg chambers hand dissected. Egg chambers were permeabilized for 30 minutes in 50 mM Tris-Cl + 188 mM NaCl + 0.1% Triton X-100 + 3% BSA, washed in PBS, then incubated for 1 hour at 37°C in 100 μl of TUNEL reaction mix according to the manufacturer’s instructions (Roche *In Situ* Cell Death Detection kit, TMR-red).

### Transmission electron microscopy

All samples were prepared for electron microscopy as described (Deng et al., 2008). 70- to 80-nm sections were stained with uranyl acetate and lead citrate and visualized using a JEOL 100CX transmission electron microscope (UCLA Brain Research Institute EM Facility). At least three individual flies of each genotype were examined for TEM studies.

### Scanning electron microscopy

Freshly sacrificed flies were mounted on their side, with one eye upward, on white tape using clear nail polish. All flies were placed on a rotating platform to permit for orientation under vacuum and were imaged as described (Gross et al., 2008).

### Female fertility tests

Single 0- to 3-day-old females were placed in a vial supplemented with dry yeast along with three sibling males and maintained at 25°C. After 4 days, the flies were removed and progeny were allowed to develop at 25°C for an additional 14 days, at which time the number of adult progeny per vial was quantified.

### S2 cell culture and transfection

S2 cells were cultured in Schneider’s Drosophila Medium (Gibco) + 10% fetal bovine serum (Invitrogen) + 1% penicillin/streptomycin (Invitrogen). For immunoprecipitations, cells were plated on 10-cm dishes at a density of 3.6×10^6^. Transfections were performed 24 hours later using the Qiagen Effectene kit according to the manufacturer’s recommendations. pMT-*lrrk-9myc* was transfected along with pAC-Gal4 and pUASp-Rab:YFP constructs (obtained from Matthew Scott) as indicated. pMT-*lrrk-9myc* expression was induced by adding 0.5 mM copper sulfate 24 hours after transfection, and cells were harvested 24 hours later.

### Lysate preparation, immunoprecipitation and western blotting

Cells were lysed in 800 ml of RIPA buffer (Upstate) containing protease inhibitor cocktail (Roche), and immunoprecipitations performed using Dynabeads from Invitrogen according to the manufacturer’s instructions. Rabbit anti-GFP (Invitrogen) or mouse anti-Myc antibodies were used for immunoprecipitation. Bound proteins were eluted at 72°C for 10 min in 1× SDS sample buffer containing 5% 2-mercaptoethanol, and were resolved on a 6% or 10% polyacrylamide gel. Proteins were transferred to an Immobilon membrane (Millipore), and labeled with mouse antibodies against Myc (Millipore, 1:10,000), GFP (Roche, 1:5000), or α-tubulin (Sigma, 1:15,000) and goat anti-mouse HRP. Blots were developed using SuperSignal West Pico Chemiluminescent Substrate (Pierce).

### Quantifications and statistical analysis

Ubiquitin-positive endosome density was calculated by manually counting the number of ubiquitin-positive puncta (labeled with FK2) per 63× field per egg chamber. Lysosome size was calculated from Lysotracker-stained samples using ImageJ software. All quantifications were performed on randomly selected egg chambers. Error bars represent standard error of the mean (s.e.m.). The two-tailed Student’s *t*-test was used to determine statistical significance.

## Supplementary material

Supplementary material available online at http://dmm.biologists.org/lookup/suppl/doi:10.1242/dmm.017020/-/DC1

Supplementary Material
