# Supplementary material for: Novel ethyl methanesulfonate (EMS)-induced null alleles of the Drosophila homolog of LRRK2 reveal a crucial role in endolysosomal functions and autophagy in vivo
Source: Dis Model Mech. 2014 Oct 2;7(12):1351–63. doi: 10.1242/dmm.017020 (PMC4257004; doi:10.1242/dmm.017020)
Supplement: Supplementary Material [file supp_7_12_1351__index.html]

Novel ethyl methanesulfonate (EMS)-induced null alleles of the Drosophila homolog of LRRK2 reveal a crucial role in endolysosomal functions and autophagy in vivo — Supplementary Material 

# Novel ethyl methanesulfonate (EMS)-induced null alleles of the *Drosophila* homolog of *LRRK2* reveal a crucial role in endolysosomal functions and autophagy *in vivo*

## DMM017020 Supplementary Material

**Files in this Data Supplement:**

- **Supplementary Material**
