# Supplementary material for: Novel ethyl methanesulfonate (EMS)-induced null alleles of the Drosophila homolog of LRRK2 reveal a crucial role in endolysosomal functions and autophagy in vivo
Source: Dis Model Mech. 2014 Oct 2;7(12):1351–63. doi: 10.1242/dmm.017020 (PMC4257004; doi:10.1242/dmm.017020)
Supplement: Supplementary Material [file supp_7.12.1351_DMM017020.pdf]

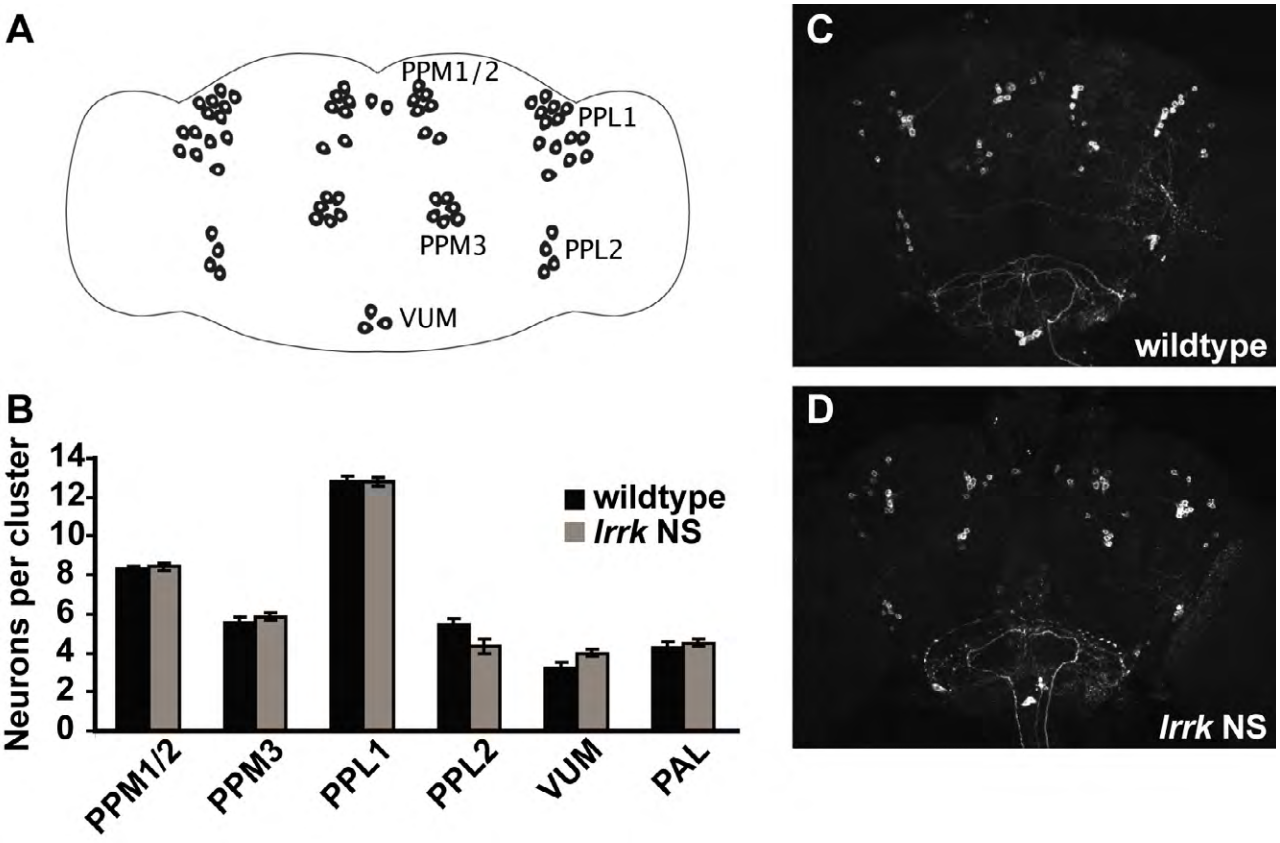


**Figure S1.** The number and gross morphology of adult dopaminergic neurons in *lrrk* NS flies are not changed with aging. (A) Schematic depicting the major dopaminergic neuron clusters in adult Drosophila brains (PPM: protocerebral posterior medial, PPL: protocerebral posterior lateral, VUM: ventral unpaired medial). The PAL cluster (protocerebral anterior lateral) is located near the opposite surface of the brain and is not depicted in this schematic. (B) Quantitation of dopaminergic neurons (as stained with anti-Tyrosine hydroxylase) in the indicated clusters from wildtype and *lrrk*$^{1/4}$ flies aged 45 days shows no significant difference in the numbers of dopaminergic neurons. (C, D) Representative images of whole brains stained with anti-Tyroxine hydroxylase from wildtype (C) and *lrrk*$^{1/4}$ (D) flies shows no significant difference in dopaminergic neuron number or morphology. In panel B, n=7 brains analyzed for wildtype, and 8 brains for *lrrk* NS.

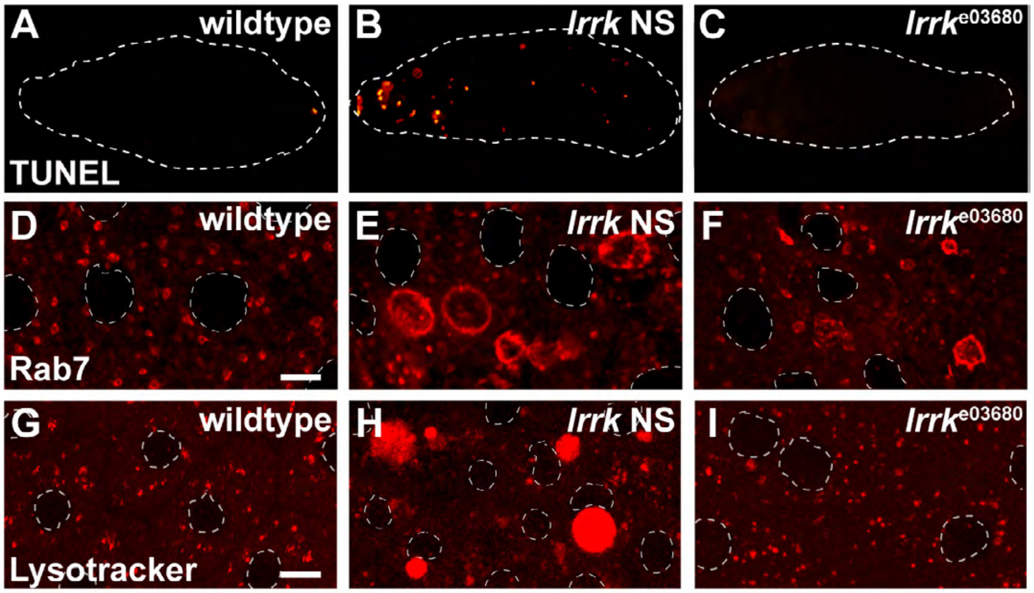


**Figure S2.** *lrrk*$^{e03680}$ flies show weak morphology defects at the Rab7-positive late endosomal compartment, and do not show lysosomal defects or premature follicle cell death observed with *lrrk* NS alleles. (A-C) TUNEL staining in wildtype (A), *lrrk* NS (B), and *lrrk*$^{e03680}$ homozygous flies (C) shows that the premature follicle cell death observed in *lrrk* NS flies is not seen in *lrrk*$^{e03680}$ homozygotes. (D-F) Rab7 staining marks the late endosomal compartment in the indicated genotypes, showing a dramatic expansion of late endosome size in *lrrk* NS follicle cells (E) versus wildtype (D), and a weaker, albeit still significant, expansion of the late endosomal compartment in a subset of *lrrk*$^{e03680}$ homozygous follicle cells (F). (G-I) Evaluation of lysosome morphology by lysotracker staining of stage 12 follicle cells from flies of the indicated genotypes. Whereas *lrrk* NS mutants (H) show a dramatic expansion of the lysosome compartment relative to wildtype (G), lysosome morphology is similar to that of wildtype in *lrrk*$^{e03680}$ homozygous flies (I). *lrrk* NS is *lrrk*$^{1/2}$. In A-C the outline of the egg chamber is marked with a dashed white line for ease of visualization. Follicle cell nuclei are marked with dashed white lines in D-I. Scale bar in D represents 5µm in D-F, and scale bar in G represents 5µm in G-I.

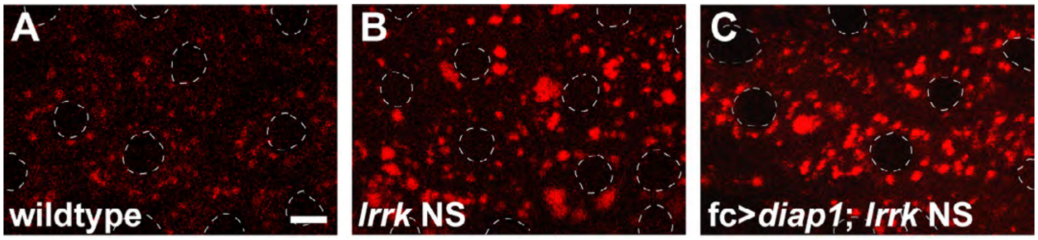


**Figure S3.** Lysosome enlargement in *lrrk* NS flies is not suppressed by expression of *diap1*. (A-C) Lysotracker staining of stage 12 follicle cells from the indicated genotypes. Expression of the caspase inhibitor *diap1* results in no significant change in the lysosome enlargement phenotype observed in *lrrk* NS cells, suggesting that lysosome enlargement does not occur downstream of caspase activation. Scale bar represents 5µm.

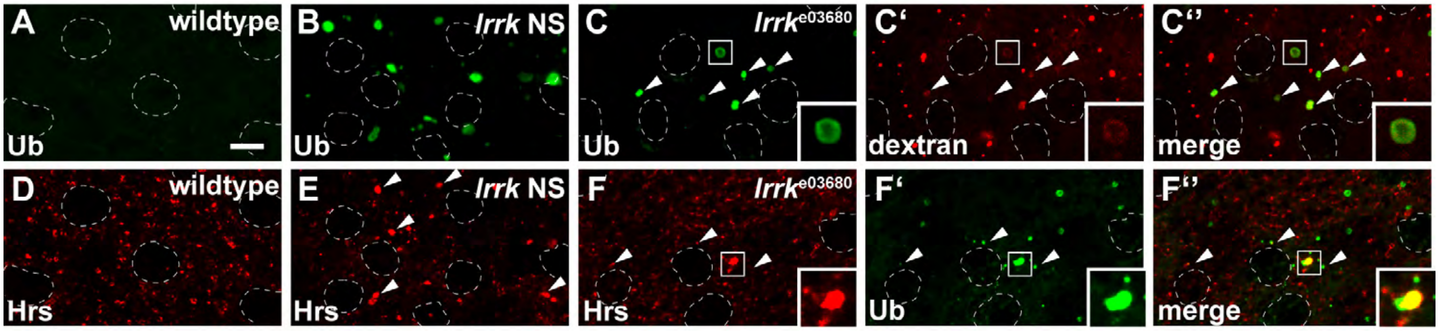


**Figure S4.** *lrrk*$^{e03680}$ flies show similar, but weaker, expansion of the early endosomal compartment and accumulation of ubiquitinated endosomal cargoes compared to *lrrk* NS flies. (A-C) Ubuquitin-protein conjugates accumulate in a concentric halo type pattern in *lrrk* NS flies (B) and to a lesser extent in *lrrk*$^{e03680}$ homozygous flies (C), but not in wildtype (A). In *lrrk*$^{e03680}$ homozygous flies, as shown earlier in *lrrk* NS flies, the ubiquitin-protein conjugates (C) colocalized with endocytosed dextran (C', C''), indicating the accumulation of ubiquitinated proteins on endosomal structures. (D-F) Labeling of early endosomes with anti-Hrs antibodies shows a weaker expansion of the anti-Hrs-positive compartment in *lrrk*$^{e03680}$ homozygous flies (F) versus *lrrk* NS (E). The ubiquitinated protein aggregates that accumulate in *lrrk*$^{e03680}$ homozygous flies (F') colocalize with Hrs (F''), suggesting that these ubiquitinated proteins are accumulating predominantly on early endosomes as is seen in *lrrk* NS flies. *lrrk* NS is *lrrk*$^{1/2}$. Follicle cell nuclei are marked with dashed white lines. Scale bar represents 5µm in all images.

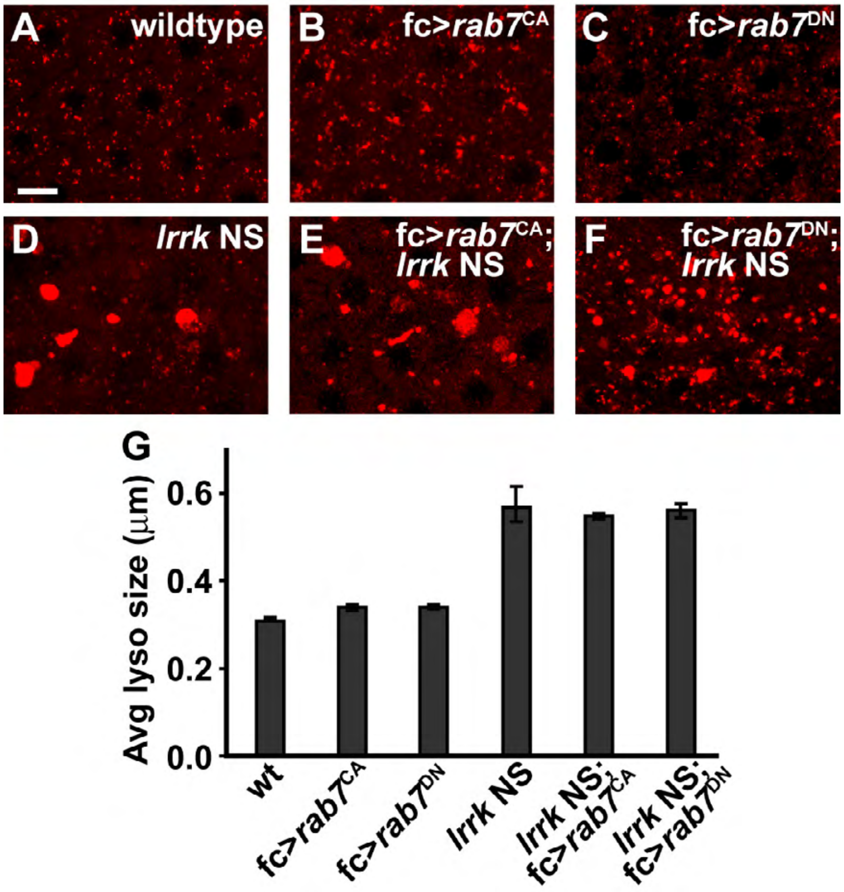


**Figure S5.** Neither constitutively active nor dominant negative Rab7 modify average lysosome size in *lrrk* NS flies. (A-F) Lysotracker stained images from stage 12 follicle cells from flies of the indicated genotypes. Relative to wildtype (A), expression of *rab7*$^{CA}$ (B) results in increased peri-nuclear clustering of lysosomes. Expression of *rab7*$^{CA}$ in the *lrrk* NS background (E) does not significantly modify the lysosome expansion phenotype seen in *lrrk* NS flies alone (D). Expression of *rab7*$^{DN}$ results in increased lysosome dispersal both in the wildtype (C), and *lrrk* NS backgrounds (F), however, lysosomes remain enlarged in *lrrk* NS flies expressing *rab7*$^{DN}$. (G) Quantification of average lysosome size based on Lysotracker staining of follicle cells from the indicated genotypes. Neither *rab7*$^{CA}$ nor *rab7*$^{DN}$ has a significant effect on average lysosome size in either the wildtype or *lrrk* NS background. Note that while lysosome enlargement appears less marked in *lrrk* NS flies expressing *rab7*$^{DN}$ (F) versus *lrrk* NS flies alone (D), this is accompanied by an increase in the number of expanded lysosomes, likely due to increased dispersal of the lysosome network. The net result is no overall change in the average lysosome size in these two genotypes, as shown in (G). The scale bar in A represents 5μm in all images. In panel G, n=8, 10, 5, 8, 7, and 5 egg chambers each respectively for the indicated genotypes.

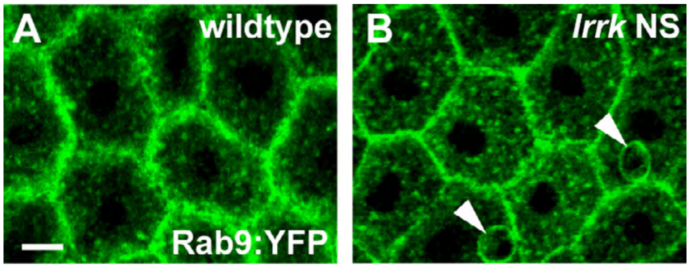


**Figure S6.** Localization of Rab9 is not affected in *lrrk* NS flies. (A, B) Wildtype (A) and *lrrk* NS (B) follicle cells expressing Rab9:YFP show no significant difference in subcellular localization of Rab9, suggesting that Lrrk does not regulate the membrane recruitment of Rab9. Note that occasional enlarged Rab9-positive structures are seen in *lrrk* NS follicle cells (arrowheads in B), which is consistent with expansion of a subset of late endosomal structures *lrrk* NS flies as we have shown earlier using Rab7. Scale bar represents 5μm.
